# The Relationship between Endorsing Gambling as an Escape and the Display of Gambling Problems

**DOI:** 10.1155/2013/156365

**Published:** 2012-12-01

**Authors:** Jeffrey N. Weatherly

**Affiliations:** Department of Psychology, University of North Dakota, Grand Forks, ND 58202-8380, USA

## Abstract

Previous research has reported a strong relationship between endorsing gambling as an escape and problem/pathological gambling as measured by the South Oaks Gambling Screen (SOGS). The present study recruited 249 university students to complete the Gambling Functional Assessment-Revised (GFA-R), which measures the function of the respondent's gambling, as well as the SOGS and the Problem Gambling Severity Index (PGSI), which was designed to identify gambling problems in the general population. Endorsing gambling as an escape on the GFA-R was again predictive of SOGS scores. The function of one's gambling was also predictive of the respondents' PGSI scores, but whether gambling for positive reinforcement or as an escape was the significant predictor differed between male and female respondents. Scores on the GFA-R subscales also accounted for a significant amount of variance in PGSI scores above and beyond that accounted for by SOGS scores. The present results support the idea that both practitioners and researchers should be interested in the function of an individual's gambling as well as the presence or the absence of pathology. They also suggest that differences in the function of gambling might also exist between the sexes.

## 1. Introduction

Problem and pathological gambling are recognized as being major societal problems, with millions of individuals suffering from them (e.g., see [1]). Because of this fact, a great deal of effort has been exerted trying to identify who might have such problems. Numerous examples of diagnostic screens can be found in the literature, including the South Oaks Gambling Screen (SOGS, [2]), the NORC DSM-IV screen for problem gamblers [3], and the Canadian Problem Gambling Index [4, 5]. The rationale behind these attempts is that if one can determine who might be experiencing problems with gambling, one is in a better position to treat, and potentially prevent, such problems.

Far less effort has been focused on an equally important issue—why people might gamble. In other words, what contingencies might be maintaining a gambler's behavior? Having such information would seem important because it seems reasonable to believe that different individuals might gamble for different reasons. It may also be the case that certain contingencies are more closely associated with gambling problems than are others. Further, it is quite possible that the reason why someone begins to gamble is different than the reason why the same person continues to gamble. Instruments designed to assess the contingencies reinforcing gambling behavior are, therefore, necessary to obtain this information.

Dixon and Johnson [6] were the first to introduce a screening instrument designed for this purpose when they forwarded the Gambling Functional Assessment (GFA). The GFA was patterned off of a similar measure designed to ascertain the contingencies maintaining self-injurious behavior [7] and was proposed to measure four potential maintaining contingencies: gambling for tangible gain, for the sensory experience, for social attention, or as an escape. Subsequent psychometric research, however, suggested that the GFA was not measuring four distinct maintaining contingencies [8]. Rather, it was only measuring two—gambling for positive reinforcement and/or as an escape—and was not cleanly parsing those two.

Because the GFA did not appear to be operating as designed, Weatherly et al. [9] revised the GFA (GFA-R) with the intention of cleanly measuring gambling maintained by positive reinforcement and escape. The GFA-R contains 16 items, each eight designed to measure gambling maintained by those two contingencies. Weatherly et al. [9] reported that the GFA-R had sound psychometric properties and cleanly parsed those two contingencies. Likewise, Weatherly et al. [10] demonstrated that the GFA-R displayed good temporal reliability and internal consistency. Overall, the research to date suggests that the psychometric properties of the GFA-R are superior to the original GFA.

In terms of the contingencies maintaining gambling behavior, research using the original GFA uncovered a potentially interesting relationship between gambling problems and endorsing gambling as an escape [8, 11]. Specifically, Miller et al. [8, 11] reported that most respondents on the GFA displayed higher scores for gambling for positive reinforcement than for gambling as an escape. However, gambling as an escape, but not gambling for positive reinforcement, was strongly predictive of potential gambling problems as measured by scores on the SOGS. Subsequent research with the GFA-R has replicated both of these findings [10, 12]. It has likewise shown that endorsing gambling as an escape is associated with both executive function and emotional regulation deficits that have been linked to gambling problems [13].

Thus, there appears to be a strong relationship between gambling problems and endorsing gambling as an escape. This relationship may not be completely surprising given that gambling as an escape is an official symptom of pathological gambling [14]. Gambling as an escape has, in fact, been a central tenant in some theories of pathological gambling (e.g., [15]). Still, what might be considered surprising is the strength of the potential relationship. For instance, Weatherly and Derenne [12] reported a correlation of 0.689 between endorsing gambling as an escape on the GFA-R and scores on the SOGS in their sample of 177 participants.

One potential criticism of such research is that it has relied on the association between the scores on the GFA-R (or the GFA) and the SOGS. Although the SOGS is a widely used screening measure within the research literature, it has been criticized on a number of fronts (e.g., see [16, 17]). One criticism is that the SOGS overestimates the prevalence of pathological gambling. A second criticism is that the SOGS was developed based on a prior, not the most recent, version of the diagnostic criteria for pathological gambling (i.e., [14]). The SOGS also focuses on the respondent's gambling history, not necessarily the severity of the issues that face the respondent. In the light of these criticisms, one cannot necessarily conclude that a strong relationship between GFA-R escape scores and SOGS scores is equivalent to predicting the severity of the respondents' gambling problems. It is possible that such a relationship might exist, but research to date has not established it beyond using the SOGS. Attempting to do so was the goal of the present study.

In the present study, respondents were recruited to complete three measures: the GFA-R, SOGS, and Problem Gambling Severity Index (PGSI), which is part of the Canadian Problem Gambling Index [4, 5]. The PGSI measures both gambling behavior and consequences and has been shown to have sound psychometric properties (e.g., [18]). Furthermore, unlike the SOGS, the PGSI was designed for the use with the general population [19].

Based on previous research, the following hypotheses were made. First, participants would endorse gambling for positive reinforcement on the GFA-R to a greater extent than they would endorse gambling as an escape. Second, endorsing gambling as an escape on the GFA-R would be a stronger predictor of SOGS scores than would be the endorsing gambling for positive reinforcement. Third, endorsing gambling as an escape on the GFA-R would be a stronger predictor of PGSI scores than would be the endorsing gambling for positive reinforcement. Finally, it was predicted that SOGS scores would be significant predictors of PGSI scores, but GFA-R escape scores would explain a significant amount of the variance in PGSI scores above and beyond that account for by SOGS scores.

## 2. Methods

### 2.1. Participants

The participants were 249 (180 females and 69 males) students enrolled in psychology courses at the University of North Dakota. The mean age of the participants was 19.8 years (SD = 3.6 years), and their self-reported grade point average was 3.4 out of 4.0 (SD = 0.5). The vast majority of the participants reported as Caucasian (229, 92.0%). All participants received (extra) course credit in their psychology class in return for their participation.

### 2.2. Materials and Procedure

Participants completed the study online using an experiment management system (SONA Systems, Ltd, Version 2.72; Tallinn, Estonia). This system guaranteed that participants could complete the materials only one time even if they were enrolled in multiple psychology courses.

The first item presented to all participants was an informed consent document that outlined the study and the participant's rights. Continuation beyond this document constituted the granting of informed consent.

After the informed consent document, participants completed four different measures. One of them was a brief demographic survey that asked them about their sex, age, grade point average, and race. They also completed the GFA-R [9], which consists of 16 items that respondents respond to on a scale that ranges from 0 (Never) to 6 (Always). Both eight-item subscales (i.e., positive reinforcement and escape) are summed to provide a score for that particular subscale. No items are reverse coded. Research has shown that the internal consistency of the GFA-R is high [10]. GFA-R scores have also been shown to have good temporal reliability (*r* = 0.80 at four weeks and *r* = 0.81 at 12 weeks [10]).

Participants also completed the SOGS [2], which consists of 20 items pertaining to the respondent's gambling history. A score of 3 or 4 suggests possible problem gambling, and a score of 5 or more suggests the probable presence of pathology. Original research [2] reported that the SOGS had high internal consistency (*α* = 0.97), and subsequent research has reported that it has fair (*α* = 0.69, [17]) to good (*α* = 0.81, [20]) internal consistency. Research has also shown that the SOGS has good temporal reliability (*r* = 0.89 at four weeks and *r* = 0.67 at 12 weeks [10]).

Finally, the participants completed the PGSI [4, 5]. The PGSI consists of 12 items, only nine of which are included when calculating respondents' scores. Research indicates that these nine items are associated with a single construct [21]. All items are answered on a four-point scale that ranges from 0 (Never) to 3 (Almost always). Scores from the nine counted items are summed, with scores of 0 indicating no gambling problems, 1-2 indicating a low level of gambling problems with few negative consequences, 3–7 indicating a moderate level of gambling problems with some negative consequences as a result, and 8 or more indicating problem gambling that includes negative consequences. Initial research on the PGSI [4] indicated that internal consistency was good (*α* = 0.84), and subsequent research (e.g., [19]) has replicated that finding. Ferris and Wynne [4] also reported that the PGSI had good temporal reliability (*r* = 0.78).

The order of presentation of the demographic form, the GFA-R, the SOGS, and the PGSI varied randomly across participants.

## 3. Results

Table 1 presents the descriptive statistics for each gambling measure. Because females constituted a majority of the sample and because the prevalence of gambling problems varies as a function of sex (see [1]), scores of the female and male participants are reported. Furthermore, 47 participants (18.9%) scored 0 on the GFA-R, suggesting that these participants either did not gamble or gambled for reasons not measured by the GFA-R (scores on the GFA-R, rather than the SOGS or PGSI, were used to make this distinction because the GFA-R was the focus of the current study. Data from participants scoring 0 on the GFA were included to provide information about the observed relationships when using a sample that may be representative of the general population, some of whom do not gamble). Thus, Table 1 also presents the descriptive statistics for the different groups when the data from these potential nongamblers were excluded.

### 3.1. Hypothesis 1

The data in Table 1 appear to support the first hypothesis; that respondents would endorse gambling for positive reinforcement to a greater extent than they would endorse gambling as an escape. When analyzing the data from the entire sample, results from Wilcoxon signed rank tests indicated that scores on the GFA-R positive reinforcement subscale were higher than scores on the escape subscale for both females (*Z* = −10.03, *P* < .001) and males (*Z* = −6.95, *P* < .001). When the nongamblers (i.e., GFA-R = 0) were excluded, scores remained significantly higher on the positive reinforcement subscale than on the escape subscale for both females (*Z *= −10.03, *P* < .001) and males (*Z *= −6.95, *P* < .001) (the *Z* values are identical for the two sets of analyses because, in the original analysis, nonresponders scored 0 on both subscales resulting in a tie score between the subscales). Thus, participants endorsed gambling for positive reinforcement to a greater extent than they did gambling as an escape. In these analyses, and all that follow, statistical significance that was considered met at *P* < .05.

### 3.2. Hypothesis 2

Table 2 presents the bivariate correlations that were observed between scores on the different measures for females and males in both the entire sample and for only those respondents who scored above 0 on the GFA-R. The correlations in Table 2 would seem to support the second hypothesis in that, in all cases, stronger correlations were observed between GFA-R escape subscales scores and SOGS scores than were observed between GFA-R positive reinforcement subscale scores and SOGS scores. These differences, however, were not always statistically significant. Tests of differences between two nonindependent *r*s [22] indicated that the correlation between the scores on the GFA-R escape subscale and on the SOGS was significantly stronger than the correlation between the GFA-R positive reinforcement subscale and the SOGS for both female samples. However, the correlations were not significantly different for either male sample. Thus, stronger correlations were observed between endorsing gambling as an escape and SOGS scores than between endorsing gambling for positive reinforcement and SOGS scores for the female respondents, but not for the male respondents.

One could argue that these results are influenced by the fact that both GFA-R escape subscale scores and SOGS scores were positively skewed. To assess this possibility, GFA-R escape subscale scores were transformed into a categorical variable, with scores of 0 coded as 0, scores between 1 and 5 coded as 1, and scores of 6 or more coded as 2 (these categories were informed by previous research [11, 13]). SOGS scores were similarly coded into a categorical variable, with scores between 0 and 2 coded as 0, between 3 and 4 coded as 1, and 5 or more coded as 2. Simultaneous linear regressions were then conducted with the transformed SOGS scores serving as the dependent measure and participants' GFA-R positive reinforcement subscale scores, which were not skewed, and the transformed GFA-R escape subscale scores serving as the potential predictor variables. Simultaneous regressions were conducted because these analyses allow for an assessment of the variance of that accounted for by each predictor variable independent of the other.

For the entire sample of female participants, the overall regression model was significant, *F*(2, 177) = 34.17, *P* < .001, and *R* ^2^ = .279. In this model, the transformed escape subscale scores were a significant predictor of the transformed SOGS scores, *β* = .449, *P* < .001, but the positive reinforcement subscales were not, *β* = .132, *P* = .076. When the data from the females who scored 0 on the GFA-R were excluded, the regression model was again significant *F*(2, 135) = 24.98, *P* < .001, and *R* ^2^ = .270. Both the escape, *β* = .434, *P* < .001, and positive reinforcement subscale scores, *β* = .168, *P* = .036, were significant predictors of SOGS scores, although the escape scores were the stronger predictor.

For the entire sample of male participants, the overall regression model was significant, *F*(2, 65 = 8.81, *P* < .001, and *R* ^2^ = .213. In this model, the transformed escape subscale scores were a significant predictor of the transformed SOGS scores, *β* = .429, *P* = .001, but the positive reinforcement subscales were not, *β* = .062, *P* = .622. When the data from the males who scored 0 on the GFA-R were excluded, the regression model was again significant *F*(2, 60) = 7.79, *P* = .001, and *R* ^2^ = .206, and escape subscale scores, *β* = .413, *P* = .002, but not positive reinforcement subscale scores, *β* = .086, *P* = .496, were significant predictors of SOGS scores.

Thus, results from these regression analyses would suggest that, for both females and males, escape scores on the GFA-R were stronger predictors of SOGS scores than were the positive reinforcement subscale scores.

### 3.3. Hypothesis 3

The correlations in Table 2 would also seem to support the third hypothesis in that, in all cases, stronger correlations were observed between GFA-R escape subscales scores and PGSI scores than were observed between GFA-R positive reinforcement subscale scores and PGSI scores. Like the correlations with the SOGS, however, these differences were not always statistically significant. Tests of differences between two nonindependent *r*s indicated that the correlation between the scores on the GFA-R escape subscale and the PGSI was significantly stronger than the correlation between the scores on the GFA-R positive reinforcement subscale and the PGSI for both female samples. The correlations were not significantly different for either male sample. Thus, as with the SOGS, whether GFA-R escape subscales scores were significantly more correlated with PGSI scores than were GFA-R positive reinforcement subscale scores varied as a function of sex.

As with the SOGS scores, PGSI scores were positively skewed (see Table 1). Thus, these scores were transformed into a categorical variable, with scores of 0 coded as 0, of between 1 and 2 coded as 1, of between 3 and 7 coded as 2, and of 8 or more coded as 3 (as suggested by [4]). A series of simultaneous linear regressions was then conducted with the transformed PGSI scores serving as the dependent measure and participants' GFA-R positive reinforcement subscale scores and their transformed GFA-R escape subscale scores serving as the predictor variables.

For the entire sample of female participants, the overall regression model was significant, *F*(2, 177) = 57.04, *P* < .001, *R* ^2^ = .392. In this model, both the transformed escape subscale scores, *β* = .522, *P* < .001, and the positive reinforcement subscales, *β* = .171, *P* = .012, were significant predictors of the transformed PGSI scores, with the escape scores being the stronger predictor. When the data from females scoring 0 on the GFA-R were excluded, the identical results were observed. The regression model was significant *F*(2, 135) = 35.72, *P* < .001, and *R* ^2^ = .346, and both the escape, *β* = .510, *P* < .001, and positive reinforcement subscale scores, *β* = .159, *P* = .036, were significant predictors of the PGSI scores, with the escape scores being the stronger predictor.

For the entire sample of male participants, the overall regression model was significant, *F*(2, 65) = 11.64, *P* < .001, *R* ^2^ = .264. In this model, both the transformed escape subscale, *β* = .244, *P* = .048, and the positive reinforcement subscales scores, *β* = .350, *P* = .005, were significant predictors of the transformed PGSI scores. In this instance, the positive reinforcement subscale scores were the stronger predictor. When the data from males scoring 0 on the GFA-R were excluded, the regression model was again significant *F*(2, 60) = 8.49, *P* = .001, and *R* ^2^ = .221. Escape subscale scores did not significantly predict PGSI scores, *β* = .240, *P* = .058, but positive reinforcement subscale scores did, *β* = .319, *P* = .013.

Thus, GFA-R subscale scores were significant predictors of PGSI scores. Which subscale was the better predictor, however, varied as a function of sex. For females, GFA-R escape subscale scores were the best predictors of  PGSI scores. For males, GFA-R positive reinforcement subscale scores were the best predictors.

### 3.4. Hypothesis 4

The SOGS was designed to identify potential pathological gamblers, whereas the PGSI was designed to test for potential gambling problems in the general population. However, one would suspect that there might be a significant overlap between the two. The high correlations between the two measures displayed in Table 2 support that contention. Hypothesis four pertains to the question of whether the GFA-R subscales are related to gambling problems (as measured by the PGSI) independent of potential pathology (as measured by the SOGS). To answer this question, multiple hierarchical linear regressions were conducted using the transformed PGSI scores as the dependent measure and transformed SOGS scores as the initial predictor. The GFA-R positive reinforcement subscale scores and the transformed GFA-R escape subscales scores were then simultaneously entered in the second block as additional predictor variables.

When the data from all female participants were subjected to this analysis, the initial model was significant, *F*(1, 178) = 194.99, *P* < .001, and *R* ^2^ = .523, and SOGS scores were a significant predictor of PGSI scores, *β* =.723, *P* < .001. When the GFA-R subscale scores were added to the analysis, the model was again significant, *F*(3, 176) = 90.11, *P* < .001, and *R* ^2^ = .606. Furthermore, the *R* ^2^ increase of .083 was statistically significant (*P* < .001). Both the SOGS, *β* = .544, *P* < .001, and the GFA-R escape subscale scores, *β* = .278, *P* < .001, were significant predictors of PGSI scores, but GFA-R positive reinforcement subscale scores were not, *β* = .100, *P* = .073. When this analysis was repeated excluding the female participants who scored 0 on the GFA-R, identical results were observed. The initial model was significant, *F*(1, 136) = 148.82, *P* < .001, and *R* ^2^ = .523, and SOGS scores were a significant predictor of PGSI scores, *β* = .723, *P* < .001. When the GFA-R subscale scores were added, the model was again significant, *F*(3, 134) = 62.93, *P* < .001, and *R* ^2^ =.585, and the *R* ^2^ increase of .062 was statistically significant (*P* < .001). SOGS, *β* = .572, *P* < .001, and GFA-R escape subscale scores, *β* = .262, *P* < .001, were significant predictors of PGSI scores, but GFA-R positive reinforcement subscale scores were not, *β* = .063, *P* = .301.

Analysis of the data from the male participants produced different results. When data from all male participants were analyzed, the initial model was significant, *F*(1, 66) = 32.68, *P* < .001, and *R* ^2^ = .331, and SOGS scores were significant predictors of PGSI scores, *β* = .575, *P* < .001. When the GFA-R subscale scores were added, the model was again significant, *F*(3, 64 = 16.61, *P* < .001, and *R* ^2^ = .438. The *R* ^2^ increase of .107 was statistically significant (*P* = .004), and SOGS scores were again significant predictors, *β* = .470, *P* < .001. However, GFA-R positive reinforcement subscale scores, *β* = .321, *P* = .004, and not GFA-R escape subscale scores, *β* = .042, *P* = .717, were significant predictors of PGSI scores. Likewise, when data from male participants scoring 0 on the GFA-R were analyzed, the initial model was again significant, *F*(1, 61) = 29.07, *P* < .001, and *R* ^2^ = .323, and SOGS scores were again significant predictors of PGSI scores, *β* = .568, *P* < .001. When the GFA-R subscale scores were added, the model was again significant, *F*(3, 59) = 13.29, *P* < .001, *R* ^2^ = .403, and the *R* ^2^ increase of .081 was statistically significant (*P* = .024). SOGS, *β* = .480, *P* < .001, and GFA-R positive reinforcement subscale scores, *β* = .278, *P* = .014, were significant predictors of PGSI scores, but GFA-R escape subscale scores were not, *β* = .042, *P* = .724.

Thus, for both female and male participants, the GFA-R subscales accounted for a significant amount of the variance in PGSI scores above and beyond that accounted for by SOGS scores. However, which subscale accounted for that increase varied as a function of sex. For females, it was endorsing gambling as an escape. For males, it was endorsing gambling for positive reinforcement.

## 4. Discussion

Previous research on the GFA-R has suggested that respondents typically endorse gambling for positive reinforcement to a greater extent that they endorse gambling as an escape, but that endorsing gambling as an escape is more strongly related to potential pathology, as measured by the SOGS, than is endorsing gambling for positive reinforcement. Results from the present study replicated the former finding; both female and male participants endorsed gambling for positive reinforcement to a significantly greater extent than they did gambling as an escape. Likewise, endorsing gambling as an escape was strongly related to potential pathology, as measured by the SOGS, for respondents of both sexes. However, endorsing gambling as an escape was only significantly more correlated with SOGS scores than endorsing gambling for positive reinforcement for the female participants.

One potential criticism of previous research is that conclusions about the GFA-R have been drawn by comparing GFA-R scores to SOGS scores. The present study, therefore, had participants complete a widely used measure designed to study potential gambling problems in the general population (i.e., the PGSI). As with the comparisons to the SOGS, GFA-R subscale scores were strongly correlated with PGSI scores. Also as with the SOGS comparisons, endorsing gambling as an escape was a significantly more correlated with PGSI scores than was endorsing gambling for positive reinforcement, but only for the female participants. In fact, for males, endorsing gambling for positive reinforcement was more predictive of PGSI scores than was endorsing gambling as an escape.

Although created to measure somewhat different things in different populations, the SOGS and PGSI scores were, not surprisingly, highly correlated. Furthermore, SOGS scores were significant predictors of PGSI scores. However, the GFA-R subscale scores accounted for a significant amount of the variance in PGSI scores above and beyond that accounted for by SOGS scores. Which GFA-R subscale accounted for that variance varied by sex. For females, endorsing gambling as an escape was the significant predictor. For males, endorsing gambling for positive reinforcement was the significant predictor. These results lead to two conclusions. First, although the GFA-R and SOGS are strongly correlated, the GFA-R is measuring something beyond what is measured by the SOGS. Second, there appears to be a significant relationship between the contingencies maintaining gambling behavior and the display of gambling problems in a university sample of participants. However, the contingency of interest appears to differ between females and males.

One might argue that given the fact that males suffer from pathological gambling significantly more frequently than females (see [1]), the results from the male participants might be most important. Before taking that tack, however, it should be noted that the display of gambling problems as measured by either the SOGS or PGSI was relatively similar for both female and male participants (see Table 1). Finding that the GFA-R was predictive of gambling problems above and beyond scores on the SOGS and that the different GFA-R subscales were differentially predictive of gambling problems between the two sexes should indicate two things to researchers and practitioners. First, there is additional information about a person's gambling behavior that can be gained by measuring the function of the behavior rather than only whether the behavior is problematic. Second, different functions of the behavior might be more indicative of gambling problems depending on the sex of the individual.

One could also argue that the sex differences reported here are consistent with the broader research. That is, research indicates that men tend to be more sensation seeking than women [23], which is consistent with the present finding that endorsing gambling for positive reinforcement was a predictor of gambling problems for male respondents. Likewise, females tend to suffer from certain disorders, such as eating disorders, more frequently than men, and those disorders have been linked to the contingencies of escape (e.g., [24, 25]). Thus, although the present results should be generalized with caution because they require replication, there does appear to be convergent validity for them within the research literature.

There are, in fact, numerous reasons to be cautious in generalizing the present results. First, the participants were all university students from the upper Midwest of the United States and were relatively young even relative to the legal age to gamble in many states. It is, for instance, possible that university students gamble for different reasons than individuals in the general population. Thus, future research should attempt to collect data from a more diverse sample. Next, the present procedure particularly targeted a largely nonpathological sample using a measure of gambling problems (i.e., the PGSI) that is the best at identifying moderate, rather than severe, gambling problems (see [18]). Thus, one cannot assume that similar results would be observed if the present procedure was conducted using a clinical sample. In fact, one might argue that a major limitation of the present study was the lack of focus on pathological gamblers. One potential counter to that argument is that identifying predictor variables of gambling problems in individuals who have yet to be diagnosed as pathological allows for the potential development of preventative measures. Then again, one cannot assume that any of the present participants will eventually become pathological gamblers.

One could also criticize the present analyses because *P *values were not adjusted to accommodate the multiple analyses that were conducted on the same data. However, it should be noted that, had such adjustments been made, the conclusions would not have changed. In the vast majority of cases, the outcomes that were statistically significant were significant at *P* < .001 and would have remained statistically significant had the threshold been raised to be more conservative.

## 5. Conclusions

The present results are largely consistent with prior findings that endorsing gambling as an escape might be indicative of the respondent experiencing gambling problems. For both sexes, endorsing gambling as an escape appears to be a strong predictor of the potential presence of problem or pathological gambling as measured by the SOGS. However, when using a more general measure of gambling problems (i.e, the PGSI), there appear to be sex differences in whether endorsing gambling for positive reinforcement (males) or as an escape (females) is more predictive of those problems. This finding is augmented by the fact that scores on the GFA-R subscales accounted for a significant amount of the variance in PGSI scores beyond that accounted for by SOGS scores. Thus, the present results lead to the conclusions that the function of one's gambling is potentially predictive of whether one might be prone to experience gambling problems. This finding is of potential importance to both practitioners and researchers. Further, finding that gambling to get something versus to get away from something might be differentially predictive of gambling problems depending on the sex of the individual highlights the need for more theoretical and empirical research in this area. It may be possible that the current results can be incorporated into existing theories of pathological gambling (e.g., [15]). Then again, new theories might be required.

## Figures and Tables

**Table 1:** Means scores on the GFA-R and its two subscales, the SOGS, and the PGSI for females, males, and all participants in either the entire sample and only for those participants who scored above 0 on the GFA-R (with standard deviations in parentheses).

Total sample					
Group (*n*)	GFA-R	GFA-R pos.	GFA-R esc	SOGS	PGSI
Females (180)	18.7 (16.4)	16.1 (13.1)	2.6 (5.9)	1.1 (1.8)	1.0 (2.6)
Males (69)	24.9 (12.5)	22.1 (10.1)	2.8 (4.0)	1.5 (1.6)	1.4 (2.4)

Total (249)	20.5 (15.6)	17.8 (12.6)	2.7 (5.4)	1.2 (1.8)	1.1 (2.5)

Respondents scoring > 0 on the GFA-R only					

Females (138)	24.4 (14.5)	21.0 (11.0)	3.4 (6.5)	1.4 (2.0)	1.3 (2.8)
Males (64)	26.8 (10.7)	23.9 (8.2)	3.0 (4.1)	1.6 (1.6)	1.5 (2.5)

Total (202)	25.2 (13.4)	21.9 (10.3)	3.3 (5.9)	1.4 (1.9)	1.4 (2.7)

**Table 2:** Bivariate correlations on the untransformed scores from each of the scales for the total sample and the subsample of respondents who scored above 0 on the GFA-R.

Total sample: females (*n* = 180)					
	GFA-R	GFA-R pos.	GFA-R esc	SOGS	PGSI
GFA-R	—	0.94**	0.67**	0.58**	0.55**
GFA-R Pos.		—	0.39**	0.43**	0.34**
GRA-R Esc.			—	0.64**	0.76**
SOGS				—	0.73**

Total sample: males (*n* = 69)					

GFA-R	—	0.96**	0.71**	0.47**	0.50**
GFA-R Pos.		—	0.47**	0.40**	0.41**
GRA-R Esc.			—	0.46**	0.54**
SOGS				—	0.72**

Respondents scoring > 0 on the GFA-R only: females (*n* = 138)					

GFA-R	—	0.90**	0.69**	0.55**	0.54**
GFA-R Pos.		—	0.31**	0.35**	0.27*
GRA-R Esc.			—	0.62**	0.75**
SOGS				—	0.73**

Respondents scoring > 0 on the GFA-R only: males (*n* = 64)					

GFA-R	—	0.94**	0.74**	0.43**	0.50**
GFA-R Pos.		—	0.46**	0.34*	0.40**
GRA-R Esc.			—	0.44**	0.52**
SOGS				—	0.72**

^∗^ *P* < .01; ^∗∗^ *P* < .001.
